# Imbalance in gut microbes from babies born to obese mothers increases gut permeability and myeloid cell adaptations that provoke obesity and NAFLD

**DOI:** 10.15698/mic2019.01.666

**Published:** 2018-12-19

**Authors:** Taylor K. Soderborg, Jacob E. Friedman

**Affiliations:** 1 Department of Pediatrics, Section of Neonatology, University of Colorado Anschutz Medical Campus, Aurora, CO USA.; 2 Department of Medicine, Division of Endocrinology, Metabolism & Diabetes, University of Colorado Anschutz Medical Campus, Aurora, CO USA.; 3 Department of Biochemistry and Molecular Genetics, University of Colorado Anschutz Medical Campus, Aurora, CO USA.

## Abstract

Non-alcoholic fatty liver disease (NAFLD) is a multifactorial disease affecting nearly 40% of obese youth and up to 10% of the general pediatric population. A key aspect of NAFLD pathogenesis is proinflammatory hepatic macrophage activation and hepatic recruitment of circulating monocytes, which originate from the bone marrow. In neonates, the activation and polarization of myeloid immune cells are normally shaped in part by systemic factors derived from intestinal microbiota during the first 1000 days of life. Perturbations of the gut microbiome, and in turn the metabolites and bacterial products released systemically, can affect the functional phenotype of these immune cells. Evidence in germ-free mice has shown that fecal microbial transfer from obese mice or obese human donors promotes obesity and inflammation in the recipients, suggesting a direct role for the gut microbiome in promoting obesity and possibly NAFLD. Indeed, patients suffering from NAFLD show evidence for dysbiosis, increased gut permeability, and changes in bile acids that drive the progression of hepatic inflammation toward non-alcoholic steatohepatitis (NASH), the more severe form of the disease. Compared with infants born to normal-weight mothers, we previously showed that the gut microbiome from neonates born to obese mothers is compositionally distinct. However, whether this alteration in early gut microbiota in infants born to obese mothers can *cause* inflammatory processes that initiate development of NAFLD or obesity is unknown. How these alterations contribute to long-term immune cell mediated liver inflammation and progression of NAFLD needs to be determined. Our recently published work (Soderborg *et al.*, Nat Commun 9:4462) demonstrates a causative role of early life microbiome dysbiosis in infants born to mothers with obesity in novel pathways that promote developmental programming of NAFLD.

At two weeks of life, the gut microbiome of infants born to obese mothers had decreased Gammaproteobacteria, an aerobic, lipopolysaccharide (LPS)-producing, pioneering microbe key for immune cell priming. Early activation of the immune system by LPS is hypothesized to be critical for proper immunological functioning later in life. Infants of obese mothers also demonstrated elevated stool levels of the microbial short-chain fatty acid metabolite butyrate, which is known to dampen macrophage inflammatory responses. This may be the result of an elevation in a butyrate-producing class of microbes such as Clostridia. We found that colonization of germ-free mice with this altered stool microbial population from two-week-old infants born to obese mothers (Inf-ObMB) is sufficient to induce a leaky gut with bacterial translocation and liver inflammation compared with mice colonized with stool from infants born to normal-weight mothers (Inf-NWMB). Most interestingly, the bacterial phagocytosis activity of bone marrow-derived macrophages from Inf-ObMB mice was reduced, as was their cytokine production in response to LPS stimulation *in vitro* compared with Inf-NWMB mice. These changes were found in macrophages derived from bone marrow following seven days *in culture*, which suggests a programmed change at the level of the bone marrow, perhaps in the hematopoietic stem cells. Identification of epigenetic changes induced by these microbial differences will provide key insight to the mechanism by which the function of these cells is altered by early life dysbiosis. The decreased response of these cells in the context of an inflamed liver, likely the result of translocated bacteria, results in unresolved inflammation, and if persistent can increase susceptibility to fatty liver and obesity. Indeed, when these mice were challenged with a Western-style diet, the Inf-ObMB-colonized mice showed increased fat mass, body weight, and liver triglycerides compared with Inf-NWMB-colonized mice, despite a lack of persistent microbiota or short-chain fatty acid differences post Western-style diet exposure. Notably, NAFLD is normally preceded by obesity, and the classic paradigm for its progression is an initial accumulation of hepatic fat followed by development of inflammation, ultimately contributing to NASH and fibrosis. Based on our results, gut leakiness and reprogramming of macrophages toward a poorly responsive phenotype play an important role in the initiation of hepatic inflammation that can also affect lipid accumulation in the liver and drive increased weight gain. Our findings are the first to suggest that early gut dysbiosis has a causal, rather than associative, role in the disorders contributing to NAFLD and obesity in infants born to obese mothers (**Fig. 1**).

**Figure 1:**
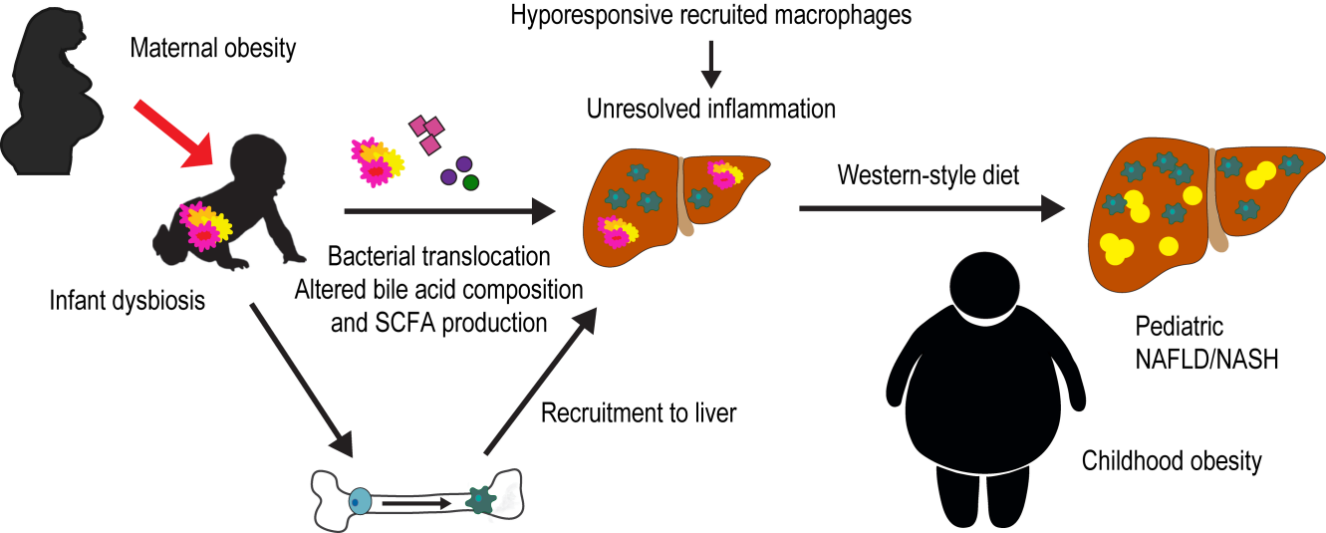
FIGURE 1: Proposed mechanism by which dysbiosis in infants of obese mothers predisposes offspring to non-alcoholic fatty liver disease (NAFLD) and obesity. Altered gut microbes in the infant of an obese mother cause altered bile acid metabolism, increased short-chain fatty acid production and a leaky gut with bacterial translocation. In the bone, infant dysbiosis results in poorly functioning recruited macrophages. In the liver, the inflammation caused by translocated bacteria remains unresolved due to hyporesponsive recruited macrophages. Exposure to a secondary hit, such as a Western-style diet, results in hepatic steatosis and obesity.

Interestingly, at the time of NAFLD diagnosis, pediatric patients often meet criteria for NASH. This suggests that inflammation and a concomitant rapid progression to fibrosis has a greater role in pediatric NAFLD compared with adult NAFLD. Furthermore, in cases of pediatric NAFLD, a periportal pattern of inflammation is more commonly reported, rather than the pericentral pattern normally seen in adult NAFLD. Metabolites and bacterial products from the microbiome travel directly to the liver via the portal vein and would therefore be a likely contributor to this unique periportal inflammation. In our study, Inf-ObMB-colonized mice had increased periportal inflammation compared with Inf-NWMB mice, which correlated with an increased modified pediatric NAFLD histological score. Livers stained with picrosirius red, published here, highlight increased collagen deposition around the portal triad in the livers of mice colonized with Inf-ObMB (**Fig. 2**). This finding suggests that neonatal microbiome dysbiosis promotes early hepatic changes that contribute to fibrosis seen in pediatric NASH.

**Figure 2:**
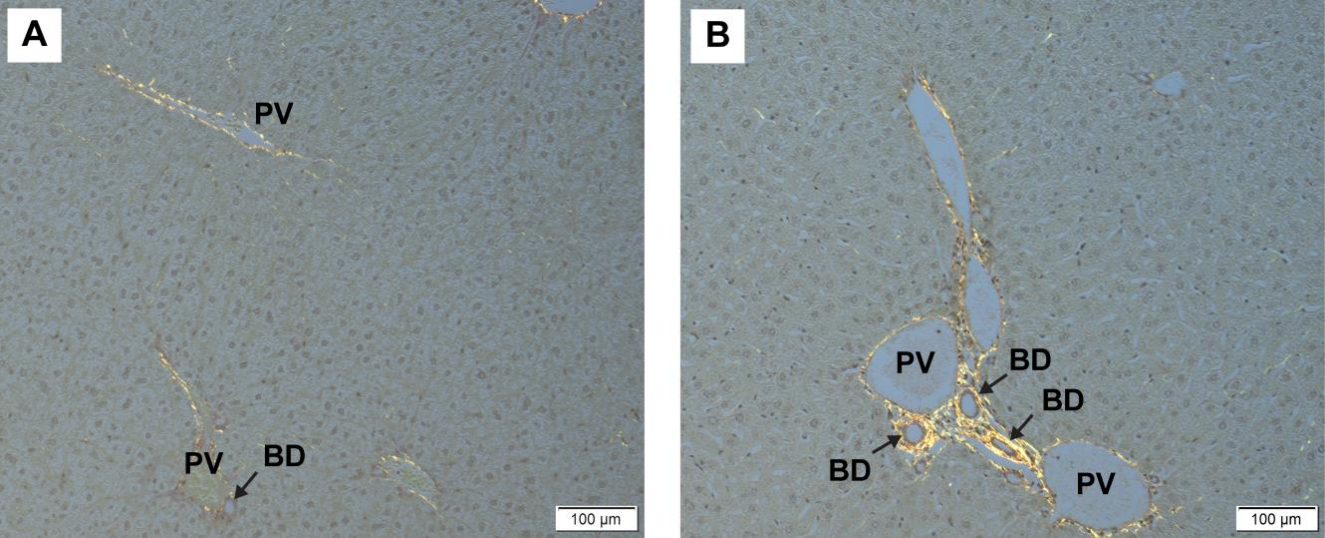
FIGURE 2: Representative photomicrographs of picrosirius red staining in the livers from mice colonized with stool from 2-week-old infants born to (A) normal-weight and (B) obese mothers. Black arrows indicate bile ducts. BD, bile duct; PV, portal vein. Sections of liver were collected and fixed in 10% formaldehyde and transferred to 70% ethanol until embedded in paraffin and processed on slides for picrosirius red staining. All staining was completed by the University of Colorado Cancer Center Research Histology Shared Resource. Images were captured on an Olympus BX53 microscope using cellSens software and DP27 camera (Olympus). All animal studies were approved by University of Colorado Institutional Animal Care and Use Committee (protocol number 00309).

Another critical role of the microbiome that we explored is its role in enterohepatic circulation of bile acids. Gut microbes are critical in the metabolism of primary bile acids into secondary bile acids. Mice colonized with Inf-ObMB showed a loss of bile acids in the feces, with compensatory changes in expression of liver genes related to bile acid synthesis, export, and import. These data, specifically the increase in bile acid receptor *Fxr* gene expression without the concomitant increase in *Shp*, its downstream target, suggest a role of early dysbiosis in impairment of the FXR-SHP pathway, which has been reported in NAFLD and warrants further exploration. This, along with the notable differences in collagen accumulation around the hepatic bile duct reported above, suggests that altered enterohepatic circulation following early life dysbiosis is an important facet of pediatric NAFLD/NASH. Further studies on the microbes responsible for the altered metabolism of bile acids in mice colonized with Inf-ObMB are warranted to help understand the development of pediatric NAFLD.

A crucial strength of our study design is the strict inclusion criteria for infants from whom stool samples were used for colonization of the germ-free mice. The stool used in this study came from babies born vaginally and exclusively breastfed. In addition, mothers had gestational weight gain within the recommended guidelines and did not receive antibiotics except in the immediate peripartum period. However, it is imperative to note that in the clinical setting, these criteria are often not met. Obesity is associated with increased cesarean section delivery and difficulty with lactation, both of which can alter offspring microbiome composition. Ultimately, we must understand the role each of these common early life influencers, independently and combined, play in altering the neonatal microbiome and the contribution gut dysbiosis has to increasing NAFLD and obesity risk. Identifying and targeting early microbial differences in the newborn infant from obese mothers could be a promising approach in the prevention of future obesity and NAFLD risk in these offspring.

